# The Book World of Medicine and Science

**Published:** 1898-06-18

**Authors:** 


### The Book World of Medicine and Science.

Mammalian Anatomy, a Preparation for Human and Comparative Anatomy. By Horace Jayne, Ph.D., M.D., Director of the Wistar Institute of Anatomy and Zoology, Professor of Zoology in the University of Pennsylvania, &c. Part I.—The Skeleton of the Cat compared with the Skeleton of Man. (London and Philadelphia. 1898. Lippincott. Pp. xix. 816, with 611 illustrations. 21s. nett.)

Professor Jayne's reputation as a sound and accomplished zoologist is so well established that any work by him must necessarily command the attention of scientific men, and the present handsome volume is of unusual importance, not alone for the facts which it embodies and their manner of presentation, but also as a contribution to the scientific teaching of comparative anatomy. It constitutes, in short, a well-nigh perfect exposition of the type system as applied to the study of the Mammalia. The influence of Huxley has popularised among English-speaking morphologists the method of teaching zoology by means of the full and accurate description of carefully selected types representative of the principal groups of the animal kingdom. Once these have been mastered, the variations from the typical structure shown by allied forms are easily recognised, and so a complete system can be readily built up. The chief merit of this plan is the way in which it dovetails with practical teaching. The types are selected not necessarily on account of their archaic nature, but because they are easy to obtain and at the same time approach completeness in the presentation of the final characters of the group to which they belong. It is for these reasons that Professor Jayne has chosen the cat as the starting point for the study of mammalian anatomy. The present work is confined to the description of the skeleton, and it may fairly be said that, as far as it goes, it does for the mammal what Huxley's "Crayfish" did for the crustacean, Ecker's "Frog" for the amphibian, and Kleinenberg's "Hydra" for the cœlenterate. Its clear style and comprehensive nature adapt it for the private student commencing the subject of zoology alone and without expert guidance; while its scientific accuracy and breadth of view render it an efficient introduction to the complex and fascinating study of mammalian morphology. At the same time each individual bone is not only described on its own account but carefully compared with the corresponding structure in the human subject, so that the book forms a thoroughly practical aid to the study of human anatomy. The description of a bone includes, to quote Professor Jayne's own words, "an explanation of its name, the areas for muscular attachment, its articulations, rules for rapid identification, the centres of ossification from which it is developed, its growth, and its variations." All these are given explicitly, succinctly, and, as far as we have been able to test them, with absolute accuracy. The most conspicuous feature of the book is its magnificent series of illustrations. The whole manner in which the book is produced reflects the greatest credit upon both author and publishers, and the student is indeed fortunate who commences the study of morphology by means of this manual. We await with great interest the further instalments of the series; if they bear out the promise of its commencement they will form an achievement of which American biological science may well be proud.

The Psychology of the Emotions. By Th. Ribot, Professor at the College of France. (London: 1897. Walter Scott. Pp. xix., 445. Price 6s. Contemporary Science Series.)

Of the three primary divisions of psychology, dealing respectively with intellect, feeling, and will, the second has of late years received by far the least attention. The psychology of feeling seemed indeed to have stagnated till interest in it was revived by the works of W. James and Lange, and further stimulated by the pathological investigations of Krafft-Ebing, Lombroso, and Moll. Till now a systematic discussion of its problems in the light of modern science was much needed, and it was to fill this gap that the present treatise was written. True it is the book can never become "popular," a word which is approaching more and more to the cognate "vulgar"; this is precluded by the difficulty of the subject. But the trained psychologist and even the ordinary thinking man—if the genus be not extinct—will arise from the perusal of Professor Ribot's essay with a wider view and a closer grasp of the problems it elucidates, and even if he is not convinced by the arguments will be compelled to strengthen his own intellectual position in reply. For, to borrow an expressive term from American politics, the author is no mugwump, sitting on the fence and juggling with well-nigh impalpable weights. He takes up a definite position in favour of the physiological theory of emotion as distinguished from that of the intellectualist; he states the reasons for his belief cogently, clearly, and yet with temperance; and he is scrupulously fair to the opposite party. The book opens with an introduction on the evolution of the affective life, which is treated from the physiological standpoint. The development of the emotions is detailed in their order—fear, anger, affection, personal emotions (*amour propre*), and sexual emotions; to joy and grief the author refuses a place in the scale of simple feelings, preferring to regard them as compounded of more primitive affective states. The general psychology of the emotions is next passed under review, commencing with pain, physical and moral, and passing through pleasure to the memory of feelings and the association of ideas. The rest of the book is devoted to the analysis of the special psychology of the individual emotions reacting from fear and the instinct of conservation to the æsthetic and intellectual sentiments. Finally, abnormal states of feeling and the decay of the affective life are considered. In the last chapter Professor Ribot adduces arguments in favour of his view that of all psychic states the feelings are the first to develop. This is not the place to discuss these views; it will suffice to remind the reader that the precedence of cognition is still maintained by many undoubted authorities. It will be gathered from what has been said that Professor Ribot's work is not "easy reading," but those who set themselves to master it will, without doubt, derive much benefit from a book which is a credit both to its author and to the series to which it belongs.

